# Association between dietary preferences and asthma among first‐grade primary school children

**DOI:** 10.1002/ped4.70021

**Published:** 2025-09-04

**Authors:** Xinyue Zhang, Xiaosa Wen, Tao Liu, Yongquan Yu, Liujie Zhu, Yingzi Chen, Dan Yu, Su Xu, Zengliang Ruan

**Affiliations:** ^1^ Department of Epidemiology and Biostatistics Key Laboratory of Environmental Medicine and Engineering of the Ministry of Education, School of Public Health Southeast University Nanjing China; ^2^ Minhang District Centre for Disease Control and Prevention Shanghai China; ^3^ Department of Biochemistry and Molecular Biology School of Medicine, Southeast University Nanjing China; ^4^ Department of Critical Care Medicine Jiangsu Provincial Key Laboratory of Critical Care Medicine, Zhongda Hospital, School of Medicine, Southeast University Nanjing China; ^5^ Department of Medical Epidemiology and Biostatistics Karolinska Institutet Stockholm Sweden

**Keywords:** Asthma, Children, Cross‐sectional study, Dietary, Primary school

## Abstract

**Importance:**

Asthma is a chronic condition characterized by airway inflammation. Dietary habits have been associated with allergic respiratory disorders, but the specific dietary preferences linked to childhood asthma remain unclear.

**Objective:**

To investigate the relationship between dietary preferences and childhood asthma in Minhang District, Shanghai, China.

**Methods:**

This cross‐sectional study was conducted in 13 sub‐districts/towns of Minhang District, Shanghai, in 2014. First‐grade primary school students were surveyed during their annual physical examinations. Data on dietary preferences and health status were collected through questionnaires completed by the children's primary caregivers. Logistic regression analysis was used to assess the relationship between dietary preferences and asthma. Stratified analyses were conducted by sex and body mass index groups.

**Results:**

A total of 8412 children, with an average age of 6.64 ± 0.29 years, participated in the survey. After adjusting for potential confounders, children who reported a preference for pickled and smoked foods exhibited a significantly elevated risk of asthma (odds ratio [OR] = 1.81; 95% confidence interval [CI]: 1.16–2.82). Subgroup analysis further revealed that girls with a preference for fried foods were at a higher risk of developing asthma (OR = 14.72; 95% CI: 1.89–114.65).

**Interpretation:**

Our findings suggest that certain dietary preferences, particularly for pickled and smoked foods, may be significant risk factors for childhood asthma.

## Graphical abstract

The relationship between dietary habits and childhood asthma is an important area of research. Our results highlight the significance of a healthy diet in the management of children's health, particularly in the prevention of asthma. Schools and families should prioritize providing a balanced and nutritious diet to promote the overall health of children.

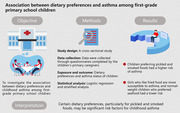

## INTRODUCTION

Asthma is a chronic respiratory disease of the airways, characterized by intermittent airflow obstruction and symptoms including wheezing, coughing, chest tightness, and shortness of breath.^1^, ^2^, ^3^ Globally, childhood asthma poses a significant health burden, affecting approximately 95.7 million children in 2021, with an age‐standardized prevalence rate of 4757.84 per 100 000 population, according to the Global Burden of Disease 2021 study.^4^ Childhood asthma has become a significant global health concern, contributing to substantial health burdens, diminished quality of life, and emotional and economic stress for children, families, and communities.^5^ In China, the prevalence of childhood asthma has risen sharply in recent decades, with the frequency of severe asthma exacerbations putting considerable pressure on pediatric healthcare systems in major cities. Nationwide epidemiological surveys have revealed a marked increase in childhood asthma prevalence in China, rising from 0.98% in 1990 to 1.97% in 2000 and further to 3.02% in 2010.^6^, ^7^ More recent regional studies, such as a 2021 cross‐sectional study in a preschool population, reported an even higher prevalence of 14.6%,^8^ suggesting an accelerating trend. These findings highlight the growing public health burden of childhood asthma and emphasize the need for targeted preventive measures.

The development of asthma is a multifactorial process influenced by genetic predispositions and environmental exposures, including dietary patterns.^9^, ^10^, ^11^, ^12^ Population‐based studies have increasingly highlighted the role of dietary habits in asthma onset and severity, with evidence suggesting that dietary choices may modify asthma risk at the population level.^13^, ^14^, ^15^, ^16^, ^17^, ^18^ For instance, large‐scale epidemiological surveys indicate that children consuming a ‘Western’ diet, characterized by high intake of processed foods, added sugars, and omega‐6 fatty acids, exhibit a higher prevalence of asthma compared to those adhering to healthier dietary patterns.^19^ These findings are supported by cohort studies linking excessive salt intake to increased asthma exacerbations and reduced lung function in pediatric populations.^20^, ^21^ Conversely, population‐based research on the Mediterranean diet has demonstrated associations between adherence to this anti‐inflammatory dietary pattern and improved asthma control.^22^ Despite these advances, gaps remain in understanding how dietary variations influence pediatric asthma outcomes, underscoring the need for further population‐level investigations to inform targeted dietary interventions.

Therefore, this cross‐sectional study aims to explore the relationship between dietary preferences and childhood asthma in Shanghai, China. By understanding these associations, we can potentially develop effective strategies for preventing and managing childhood asthma.

## METHODS

### Ethical approval

The study was approved by the Ethics Committee of Minhang District Center for Disease Control and Prevention (approval number: EC‐P‐2014‐006) and adhered to the Declaration of Helsinki. Written informed consent was obtained from the primary caregivers of all participants.

### Study design and population

**FIGURE 1:**
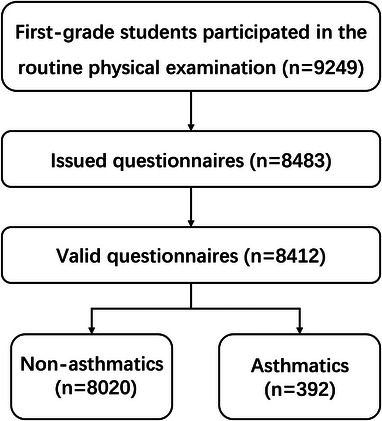
Flowchart of the study.

This was a cross‐sectional analysis using data from the first wave of the Minhang Pediatric Biobank (MPB) cohort study, a longitudinal research project investigating genetic and environmental risk factors for childhood disease.^23^ The survey was conducted during the 2014–2015 school year, enrolling first‐grade students from 42 public elementary schools in 13 sub‐districts/towns of Minhang, a district located in the southwest of downtown Shanghai (Figure 1). We selected this district as the study location because it represents the urban‐suburban transitional zone in Shanghai with diverse socioeconomic demographics. Importantly, it offers unique advantages for enabling comprehensive analysis through its capacity to integrate detailed dietary surveys with personal health records. In addition, its food culture incorporates both traditional Shanghainese cuisine and modern dietary trends, offering valuable insights into dietary effects.

### Data collection

Primary caregivers of the children were engaged in face‐to‐face interviews to fill out a detailed questionnaire. The questionnaire collected information on the parents’ education level, children's demographic characteristics (sex, age), lifestyle factors (e.g., frequency of breakfast, snacks, and dietary preferences), and medical history. Additionally, children underwent physical examinations to measure height, weight, and other relevant parameters. We implemented a barcode scanning system to ensure data quality control throughout participant enrollment, questionnaire administration, and physical measurements. Each participant was assigned a unique barcode linked to their questionnaire, which was scanned at every interaction point to enable cross‐verification of identity across data sources and prevent transcription errors before data entry. The implementation of this system has significantly improved the accuracy and efficiency of data collection and management.

### Ascertainment of children's dietary preferences and asthma

Children's dietary preferences we categorized into three levels: like, average, and dislike, based on reports from their primary caregivers. The questionnaire assessed preferences for 12 food categories, including vegetables, fruits, meat, poultry, soy products, seafood, dairy products, eggs, pickled and smoked foods, confectionery, fried foods, and puffed foods. Asthma status was determined based on caregivers’ reports. The potential confounding factors included the child's sex, age, dietary habits, breakfast frequency, fast food consumption, snacking habits, picky eating behaviors, and the parents’ education level.

### Statistical analysis

Descriptive statistics were employed to characterize the study population. Categorical variables are presented as frequencies (percentages). In addition, as the continuous variables (age, height, weight, and body mass index) demonstrated approximate normality, they are all reported as mean (standard deviation). The association between dietary preference and asthma was assessed using logistic regression models, with or without adjusting for confounders such as age, sex, body mass index (BMI), parental education level, fast food consumption, and snacking habits. In the models, children's asthma status was the dependent variable, and dietary preferences were the independent variable, using the ‘dislike’ group as the reference category. The results are presented as odds ratios (ORs) with 95% confidence intervals (CIs). Stratified analyses were performed to examine potential effect modification by sex and BMI groups. In this study, BMI was used as the primary indicator for assessing overweight or obesity status in children. For stratified analyses, BMI classification adhered to the age‐ and sex‐specific cutoff points established by the Chinese National Health Commission, which define overweight as ≥85^th^ percentile and obesity as ≥95^th^ percentile of the BMI‐for‐age distribution, ensuring consistency with both international and national guidelines. This standard (WS/T 586‐2018)^24^, ^25^ was jointly developed by the Institute of Child and Adolescent Health at Peking University and the National Institute for Nutrition and Health of the Chinese Center for Disease Control and Prevention, which was officially issued by the National Health Commission of the People's Republic of China. All statistical analyses were conducted using R software version 4.3.2, applying a significance threshold of *P* < 0.05 for two‐tailed tests.

## RESULTS

This survey included a total of 8412 students and their parents from 13 sub‐districts/towns. Among the participants, 392 children were reported to have asthma, while 8020 were not. The sample consisted of 4339 boys (51.58%) and 4073 girls (48.42%), with a mean age of 6.64 ± 0.29 years. The overall mean BMI for all children was 16.27 ± 2.54 kg/m^2^. In terms of parental education, 3494 children (41.54%) had mothers who had completed university education (including graduate studies), while 3980 children (47.31%) had fathers with a university‐level education. Table 1 provides further details on the characteristics of the children.

**TABLE 1:** Demographic characteristics of the participants

Characteristics	Asthmatics	Non‐asthmatics	Total
Age (years)	6.65 ± 0.28	6.64 ± 0.29	6.64 ± 0.29
Height (cm)	124.90 ± 5.15	123.84 ± 5.40	123.89 ± 5.39
Weight (kg)	26.20 ± 5.39	25.10 ± 5.16	25.15 ± 5.17
Body mass index (kg/m^2^)	16.66 ± 2.57	16.25 ± 2.53	16.27 ± 2.54
Sex			
Male	270 (3.21)	4069 (48.37)	4339 (51.58)
Female	122 (1.45)	3951 (46.97)	4073 (48.42)
Mother's educational level			
Elementary School and above	3 (0.04)	80 (0.95)	83 (0.99)
Junior High School	28 (0.33)	684 (8.13)	712 (8.46)
Senior High School/Technical Secondary School	71 (0.84)	1526 (18.14)	1597 (18.98)
Junior College	97 (1.15)	2359 (28.04)	2456 (29.20)
Bachelor's (include Master's)	192 (2.28)	3302 (39.25)	3494 (41.54)
Father's educational level			
Elementary School and above	1 (0.01)	37 (0.44)	38 (0.45)
Junior High School	24 (0.29)	443 (5.27)	467 (5.55)
Senior High School/Technical Secondary School	73 (0.87)	1592 (18.93)	1665 (19.79)
Junior College	93 (1.11)	2116 (25.15)	2209 (26.26)
Bachelor's (include Master's)	199 (2.37)	3781 (44.95)	3980 (47.31)
Eat fast food			
4–7 times a week	4 (0.05)	99 (1.18)	103 (1.22)
1–3 times a week	16 (0.19)	299 (3.55)	315 (3.74)
1–3 times a month	173 (2.06)	3639 (43.26)	3812 (45.32)
<1 time per month	196 (2.33)	3886 (46.20)	4082 (48.53)
Snacking			
Never	2 (0.02)	72 (0.86)	74 (0.88)
Seldom	110 (1.31)	2174 (25.84)	2284 (27.15)
Sometimes	177 (2.10)	3819 (45.40)	3996 (47.50)
Often	100 (1.19)	1872 (22.25)	1972 (23.44)
Vegetables			
Dislike	38 (0.45)	647 (7.69)	685 (8.14)
Average	219 (2.60)	4619 (54.91)	4838 (57.51)
Like	134 (1.59)	2671 (31.75)	2805 (33.35)
Fruit			
Dislike	7 (0.08)	109 (1.30)	116 (1.38)
Average	87 (1.03)	2273 (27.02)	2360 (28.06)
Like	296 (3.52)	5555 (66.04)	5851 (69.56)
Meat			
Dislike	14 (0.17)	378 (4.49)	392 (4.66)
Average	122 (1.45)	2861 (34.01)	2983 (35.46)
Like	255 (3.03)	4691 (55.77)	4946 (58.80)
Poultry			
Dislike	22 (0.26)	564 (6.70)	586 (6.97)
Average	168 (2.00)	3777 (44.90)	3945 (46.90)
Like	200 (2.38)	3561 (42.33)	3761 (44.71)
Soybean			
Dislike	47 (0.56)	815 (9.69)	862 (10.25)
Average	194 (2.31)	4197 (49.89)	4391 (52.20)
Like	150 (1.78)	2902 (34.50)	3052 (36.28)
Seafood			
Dislike	77 (0.92)	1354 (16.10)	1431 (17.01)
Average	157 (1.87)	3501 (41.62)	3658 (43.49)
Like	156 (1.85)	3054 (36.31)	3210 (38.16)
Dairy			
Dislike	11 (0.13)	222 (2.64)	233 (2.77)
Average	96 (1.14)	2380 (28.29)	2476 (29.43)
Like	284 (3.38)	5316 (63.20)	5600 (66.57)
Egg			
Dislike	22 (0.26)	432 (5.14)	454 (5.40)
Average	162 (1.93)	3485 (41.43)	3647 (43.35)
Like	206 (2.45)	4002 (47.57)	4208 (50.02)
Pickled and smoked			
Dislike	232 (2.76)	4968 (59.06)	5200 (61.82)
Average	126 (1.50)	2473 (29.40)	2599 (30.90)
Like	33 (0.39)	436 (5.18)	469 (5.58)
Confectionery			
Dislike	21 (0.25)	407 (4.84)	428 (5.09)
Average	121 (1.44)	2664 (31.67)	2785 (33.11)
Like	249 (2.96)	4844 (57.58)	5093 (60.54)
Fried foods			
Dislike	27 (0.32)	755 (8.98)	782 (9.30)
Average	182 (2.16)	3904 (46.41)	4086 (48.57)
Like	180 (2.14)	3252 (38.66)	3432 (40.80)
Puffed food			
Dislike	30 (0.36)	711 (8.45)	741 (8.81)
Average	157 (1.87)	3626 (43.11)	3783 (44.97)
Like	203 (2.41)	3574 (42.49)	3777 (44.90)

Data are presented as mean ± standard deviation or *n* (%).

Missing (*n*): Mother's educational level (70); Father's educational level (53); Eat fast food (100); Snacking (86); Vegetables (84); Fruit (85); Meat (91); Poultry (120); Soybean (107); Seafood (113); Dairy (103); Egg (103); Pickled and smoked (144); Confectionery (106); Fried foods (112); Puffed food (111).

**FIGURE 2:**
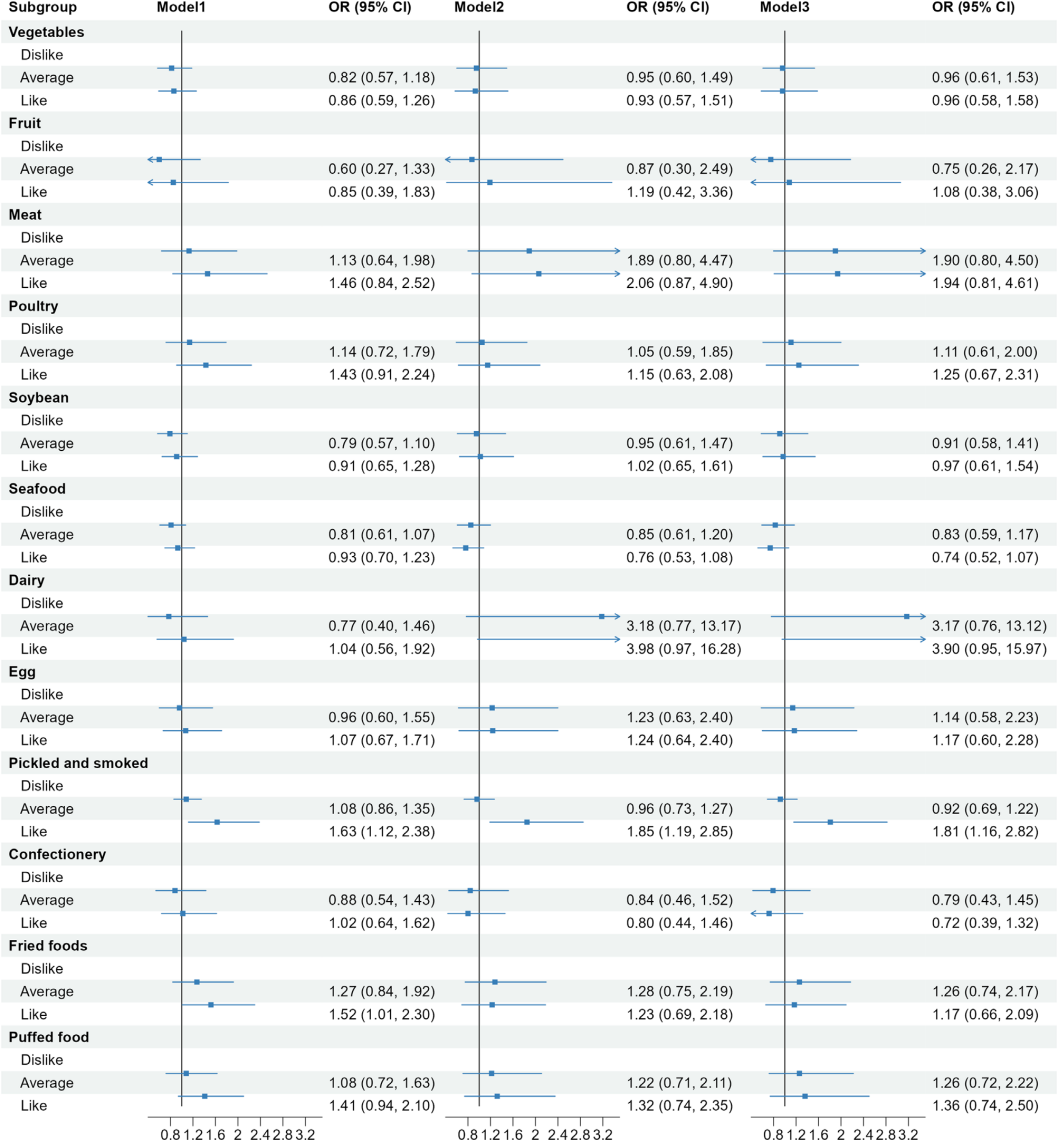
A forest plot for the association between dietary behaviors and the risk of asthma in children. The analysis utilized three models: Model 1 is the unadjusted model, Model 2 adjusts for age and gender, and Model 3 further adjusts for body mass index, parental education level, frequency of fast‐food consumption, and snacking habits. The plot illustrates the effect estimates and confidence intervals for each model. OR, odds ratio; CI, confidence interval.

Figure 2 presents the OR for the relationship between dietary preferences and asthma, with results from both unadjusted and adjusted models. In the unadjusted model, children who preferred pickled and smoked foods had higher odds of developing asthma (OR = 1.63, 95% CI: 1.12–2.38, *P* = 0.011). The association continued to show statistical significance after controlling for all selected confounding factors (OR = 1.81, 95% CI: 1.16–2.82, *P* = 0.009). Additionally, children who preferred other types of foods, including vegetables, fruits, meat, poultry, soybeans, seafood, dairy products, eggs, confectionery, as well as fried and puffed foods, did not demonstrate a significant association with the risk of asthma.

The effect size estimates for the association between dietary preferences and asthma in sex‐specific analyses after adjusting for potential confounders are shown in Table 2. Among girls, a preference for fried foods was significantly associated with an increased risk of asthma (OR = 14.72, 95% CI: 1.89–114.65, *P* = 0.010), whereas no such association was observed in boys. Conversely, boys who preferred pickled and smoked foods had a 2.01‐fold higher asthma risk compared to those who did not (OR = 2.01, 95% CI: 1.19–3.39, *P* = 0.009), but this association was not significant in girls. In the stratified analysis based on BMI categories, the results indicated that a preference for pickled and smoked foods was associated with higher asthma risk in normal‐weight children (OR = 1.89, 95% CI: 1.03–3.48, *P* = 0.041). Furthermore, we observed that normal‐weight children who favored seafood had a lower risk of developing asthma, which suggested that seafood may have a protective effect (OR = 0.60, 95% CI: 0.37–0.97, *P* = 0.036) (Table 3).

**TABLE 2:** Sex‐specific analyses on the association between dietary preference and childhood asthma

	Female		Male	
Characteristics	OR (95% CI)	*P*‐value	OR (95% CI)	*P*‐value
Vegetables				
Dislike	1.00		1.00	
Average	1.80 (0.54, 5.99)	0.339	0.86 (0.52, 1.43)	0.560
Like	2.16 (0.62, 7.54)	0.225	0.77 (0.44, 1.34)	0.351
Fruit				
Dislike	1.00		1.00	
Average	0.36 (0.07, 1.72)	0.199	1.13 (0.26, 4.93)	0.871
Like	0.39 (0.08, 1.81)	0.228	1.84 (0.43, 7.86)	0.413
Meat				
Dislike	1.00		1.00	
Average	0.91 (0.26, 3.23)	0.889	2.84 (0.85, 9.43)	0.089
Like	1.02 (0.29, 3.65)	0.971	2.80 (0.84, 9.37)	0.095
Poultry				
Dislike	1.00		1.00	
Average	1.20 (0.40, 3.55)	0.749	1.06 (0.52, 2.14)	0.876
Like	1.18 (0.38, 3.73)	0.772	1.24 (0.59, 2.60)	0.562
Soybean				
Dislike	1.00		1.00	
Average	0.94 (0.43, 2.05)	0.872	0.90 (0.53, 1.55)	0.713
Like	0.92 (0.40, 2.10)	0.844	1.01 (0.58, 1.76)	0.979
Seafood				
Dislike	1.00		1.00	
Average	0.80 (0.41, 1.55)	0.503	0.81 (0.54, 1.23)	0.324
Like	0.60 (0.30, 1.21)	0.157	0.80 (0.52, 1.22)	0.293
Dairy				
Dislike	1.00		1.00	
Average	1.28 (0.16, 9.95)	0.816	5.30 (0.72, 39.06)	0.102
Like	2.31 (0.31, 17.33)	0.414	5.65 (0.77, 41.16)	0.088
Egg				
Dislike	1.00		1.00	
Average	2.12 (0.49, 9.13)	0.313	0.88 (0.41, 1.89)	0.744
Like	2.10 (0.49, 9.04)	0.320	1.92 (0.43, 1.98)	0.837
Pickled and smoked				
Dislike	1.00		1.00	
Average	0.91 (0.55, 1.51)	0.714	0.88 (0.62, 1.25)	0.475
Like	1.45 (0.61, 3.44)	0.396	2.01 (1.19, 3.39)	0.009
Confectionery				
Dislike	1.00		1.00	
Average	1.07 (0.24, 4.73)	0.931	0.73 (0.37, 1.44)	0.368
Like	1.04 (0.24, 4.59)	0.958	0.64 (0.32, 1.27)	0.201
Fried foods				
Dislike	1.00		1.00	
Average	8.51 (1.13, 64.15)	0.038	0.81 (0.44, 1.46)	0.476
Like	14.72 (1.89, 114.65)	0.010	0.57 (0.30, 1.08)	0.086
Puffed food				
Dislike	1.00		1.00	
Average	1.32 (0.45, 3.93)	0.613	1.33 (0.68, 2.61)	0.400
Like	1.08 (0.34, 3.45)	0.893	1.60 (0.78, 3.30)	0.202

Logistic regression models are adjusted for age, body mass index, parental education level, frequency of fast‐food consumption, and snacking habits of the child.

Abbreviations: CI, confidence interval; OR, odds ratio.

**TABLE 3:** Body mass index (BMI)‐specific analyses on the association between dietary preference and childhood asthma

	Normal		Overweight and obesity	
Characteristics	OR (95% CI)	*P*‐value	OR (95% CI)	*P*‐value
Vegetables				
Dislike	1.00		1.00	
Average	1.08 (0.54, 2.14)	0.827	0.92 (0.48, 1.75)	0.799
Like	1.03 (0.50, 2.12)	0.932	0.95 (0.47, 1.93)	0.885
Fruit				
Dislike	1.00		1.00	
Average	0.74 (0.17, 3.30)	0.697	0.74 (0.16, 3.41)	0.704
Like	1.07 (0.25, 4.66)	0.923	1.06 (0.24, 4.72)	0.941
Meat				
Dislike	1.00		1.00	
Average	0.98 (0.40, 2.39)	0.956	–	–
Like	0.97 (0.39, 2.42)	0.947	–	–
Poultry				
Dislike	1.00		1.00	
Average	1.96 (0.75, 5.11)	0.169	0.69 (0.32, 1.51)	0.351
Like	2.66 (0.99, 7.20)	0.053	0.62 (0.27, 1.41)	0.254
Soybean				
Dislike	1.00		1.00	
Average	0.89 (0.49, 1.62)	0.707	0.89 (0.45, 1.73)	0.722
Like	0.90 (0.48, 1.68)	0.739	1.09 (0.55, 2.18)	0.808
Seafood				
Dislike	1.00		1.00	
Average	0.81 (0.51, 1.27)	0.350	0.87 (0.50, 1.50)	0.606
Like	0.60 (0.37, 0.97)	0.036	0.98 (0.56, 1.70)	0.940
Dairy				
Dislike	1.00		1.00	
Average	3.93 (0.53, 29.24)	0.181	2.35 (0.30, 18.37)	0.416
Like	5.22 (0.71, 38.15)	0.104	2.57 (0.34, 19.63)	0.363
Egg				
Dislike	1.00		1.00	
Average	1.07 (0.48, 2.38)	0.877	1.46 (0.43, 4.99)	0.548
Like	1.07 (0.48, 2.39)	0.874	1.50 (0.44, 5.08)	0.516
Pickled and smoked				
Dislike	1.00		1.00	
Average	0.96 (0.66, 1.40)	0.840	0.87 (0.55, 1.38)	0.559
Like	1.89 (1.03, 3.48)	0.041	1.78 (0.92, 3.44)	0.088
Confectionery				
Dislike	1.00		1.00	
Average	1.19 (0.45, 3.13)	0.722	0.57 (0.25, 1.30)	0.179
Like	1.11 (0.42, 2.91)	0.834	0.47 (0.20, 1.10)	0.082
Fried foods				
Dislike	1.00		1.00	
Average	1.02 (0.53, 1.96)	0.964	1.84 (0.71, 4.78)	0.211
Like	0.01 (0.45, 1.87)	0.806	1.73 (0.63, 4.70)	0.286
Puffed food				
Dislike	1.00		1.00	
Average	1.88 (0.81, 4.34)	0.139	0.83 (0.38, 1.83)	0.648
Like	1.76 (0.72, 4.27)	0.215	1.12 (0.48, 2.63)	0.793

Logistic regression models are adjusted for age, BMI, parental education level, frequency of fast‐food consumption, and snacking habits of the child. The missing risk estimate for meat among overweight and obese children is due to the lack of meat‐disliking subject in this subgroup.

Abbreviations: BMI, body mass index; CI, confidence interval; OR, odds ratio.

## DISCUSSION

This study utilized a cross‐sectional design to investigate the associations between children's dietary preferences and the risk of asthma among first‐grade students in Shanghai, China. Our results suggested that a preference for pickled and smoked foods might increase the risk of childhood asthma. Stratified analysis further confirmed the significant association of pickled and smoked foods with childhood asthma. It also revealed that girls with a preference for fried foods had a higher risk of developing asthma, while normal‐weight children who preferred seafood had a lower risk.

In the present study, the prevalence of asthma in first‐grade primary school children was found to be 4.66%, which is lower than the accumulated asthma prevalence of 7.57% and current prevalence in the last two‐year period of 5.73% reported for 0–14 years old children in Shanghai in 2013.^6^ This discrepancy may stem from methodological variations across studies, including differences in survey implementation, sampling strategies, and diagnostic criteria (ranging from self‐reported to physician‐diagnosed cases, whereas we relied on primary caregiver reports). Furthermore, our study specifically examined first‐grade students typically aged 6–7 years, contrasting with broader age ranges of 3–7 or 0–14 years in other studies. Given known variations in asthma prevalence across pediatric age groups, such focused sampling likely contributes to the observed differences. Additionally, Minghang represents Shanghai's urban‐suburban transition with diverse socioeconomic and migrant population demographics, but may not fully represent Shanghai's overall profile. These methodological and demographic considerations may collectively contribute to the observed prevalence variations between studies. Additionally, our study revealed that childhood asthma was significantly more prevalent in males than in females, with a male‐to‐female ratio of 2.35:1, which was consistent with previous research.^7^, ^26^, ^27^ This discrepancy could be attributed to factors such as differences in hormonal levels, respiratory responsiveness, smooth muscle tone, and airway size between boys and girls, as well as increased exposure to allergens or pathogens due to lifestyle behaviors.^1^, ^28^

Our findings indicated that certain dietary preferences, such as preferences for pickled and smoked foods, could be important contributors to the risk of asthma in children. These results were aligned with recent studies linking the intake of pickled and smoked foods with higher asthma risk in children. For example, Burney's research suggested that the rising prevalence of asthma in some regions might be associated with Western dietary patterns. These foods, often high in salt, saturated fat, and preservatives, are associated with asthma and respiratory diseases in both children and adults in England and Wales.^29^ While some studies indicated that the consumption of salt‐rich foods was associated with an increased prevalence of asthma and other respiratory diseases in adolescents and adults,^30^, ^31^ other research suggested that increased dietary sodium might have a beneficial effect on asthma, highlighting the complexity of this relationship.^32^ The significant associations of pickled and smoked foods with childhood asthma may be attributed to various pathways. First, nitrites in these foods undergo gastric conversion to N‐nitroso compounds, which may induce respiratory oxidative stress by depleting glutathione and generating reactive oxygen species.^33^ Second, polycyclic aromatic hydrocarbons, such as benzo[a]pyrene, may promote Th2‐mediated inflammation via activation of the aryl hydrocarbon receptor pathway.^34^ Third, advanced glycation end products, formed during high‐temperature processing, disrupt the airway epithelial barrier by crosslinking with their receptors.^35^ Finally, high salt intake from pickled foods may exacerbate asthma through the overactivation of epithelial sodium channels, dehydrating airway surfaces, triggering bronchoconstriction, and reducing gut‐derived short‐chain fatty acids, thereby impairing immune tolerance.^36^, ^37^

Subgroup analysis revealed a significant association between fried food preference and asthma risk in girls, though no such association was observed in boys. However, it should be noted that although the results in girls reached statistical significance, the wide confidence interval in girls suggests substantial uncertainty regarding the effect size, possibly due to limited subgroup sample size or sparse data distribution, underscoring the need for cautious interpretation and replication in larger studies. Although direct causal pathways remain unverified, given the absence of direct biochemical measurements in this study, the observed sex‐specific associations between food preference and childhood asthma may reflect sexual dimorphism in metabolic and endocrine regulation. For example, previous evidence suggests that estrogen may amplify lipid accumulation in adipose tissue, potentially exacerbating airway inflammation in females consuming high‐calorie diets.^38^, ^39^, ^40^ Conversely, sex differences in several enzyme activities (e.g., CYP3A4) have been reported to reduce xenobiotic metabolism efficiency in females.^41^ We acknowledge that these mechanisms are hypothesized based on previous studies, and future research should prioritize metabolomic profiling to disentangle these interactions.

Our results reveal a significant inverse association between seafood preference and asthma risk in children with normal body weight. This protective effect may be attributed to bioactive components in seafood, particularly omega‐3 polyunsaturated fatty acids (n‐3 PUFAs) and vitamin D. Mechanistically, n‐3 PUFAs competitively inhibit pro‐inflammatory mediators by suppressing cyclooxygenase‐2 activity,^42^ while vitamin D promotes immune tolerance through Foxp3‐mediated regulatory T‐cell differentiation.^43^ These synergistic pathways likely contribute to the observed risk reduction, offering a plausible rationale for dietary interventions in asthma prevention. Nevertheless, further research is warranted to validate these mechanisms and explore potential interactions with body weight status.

Despite these valuable insights, it is essential to acknowledge several limitations in the current study. First, as a cross‐sectional study, it inherently lacks the capacity to establish a causal relationship between the identified factors. Future research should focus on conducting prospective cohort studies with longitudinal dietary assessments and incident asthma cases to confirm these associations. Second, the study's focus on children from a relatively small area may limit the generalizability of the findings, as dietary and cultural differences may vary across regions. Third, data were primarily collected through parental questionnaires, which may introduce reporting biases and recall inaccuracies. Fourth, the data in this study were collected a decade ago, which may raise concerns about relevance. However, our focus on dietary risk factors for childhood asthma examines associations that are biologically plausible and unlikely to vary substantially over time, which is partially supported by the consistency of our results with other literature.^29^, ^44^ Fifth, dietary preferences were assessed via subjective questionnaires. While this method has inherent limitations (e.g., lack of quantitative exposure metrics), it is a pragmatic, cost‐effective approach for large‐scale surveys and aligns with methodologies employed in prior studies.^45^, ^46^, ^47^ Additionally, the current dietary classification system relies on general food group preferences rather than precise quantification of consumption levels and nutrient intakes, which precludes definitive conclusions about dose‐response relationships. This also restricts our ability to assess the direct impact of specific nutrients on asthma outcomes and highlights the need for future prospective cohort studies to incorporate standardized dietary assessment tools, such as food frequency questionnaires or 24‐hour dietary recalls, to enable more comprehensive analysis and clarify potential causal mechanisms.

In summary, we identified several dietary preferences, such as pickled and smoked foods, that correlate with an increased risk of asthma in children. Understanding these associations can help inform targeted dietary interventions for the prevention and management of asthma.

## CONFLICT OF INTEREST

The authors declare no conflict of interest.
